# *Edwardsiella tarda* Outer Membrane Protein C: An Immunogenic Protein Induces Highly Protective Effects in Flounder (*Paralichthys olivaceus*) against Edwardsiellosis

**DOI:** 10.3390/ijms17071117

**Published:** 2016-07-12

**Authors:** Fuguo Liu, Xiaoqian Tang, Xiuzhen Sheng, Jing Xing, Wenbin Zhan

**Affiliations:** 1 Laboratory of Pathology and Immunology of Aquatic Animals, Ocean University of China, 5 Yushan Road, Qingdao 266003, China; aquamedic@ouc.edu.cn (F.L.); tangxq@ouc.edu.cn (X.T.); xzsheng@ouc.edu.cn (X.S.); xingjing@ouc.edu.cn (J.X.); 2 Laboratory for Marine Fisheries Science and Food Production Processes, Qingdao National Laboratory for Marine Science and Technology, No. 1 Wenhai Road, Aoshanwei Town, Jimo, Qingdao 266071, China

**Keywords:** outer membrane protein C, *Edwardsiella tarda*, vaccine, flounder, immune response

## Abstract

Outer membrane protein C of *Edwardsiella tarda* is a major cell surface antigen and it was identified to be an immunogenic protein by Western blot using flounder (*Paralichthys olivaceus*) anti-recombinant OmpC (rOmpC), and anti-*E. tarda* antibodies. rOmpC tested the immune protective effect against *E. tarda* challenge in a flounder model and produced a relative percentage of survival rate of 85%. The immune response of flounder induced by rOmpC was investigated, and the results showed that: (1) the levels of specific serum antibodies induced by rOmpC were significantly higher than the control group after the second week after immunization, and the peak level occurred at week five after immunization; (2) rOmpC could induce the proliferation of sIg+ lymphocytes, and the peak levels of sIg+ lymphocytes in blood, spleen, and pronephros occurred at 4–5 weeks after immunization; and (3) the *MHCIIα*, *CD4-1*, *IL-1β*, *IL-6* and *TNF-α* genes were significantly induced after being injected with rOmpC. Taken together, these results demonstrated that rOmpC could evoke highly protective effects against *E. tarda* challenge and induce strong innate immune response and humoral immune response of flounder, which indicated that OmpC was a promising vaccine candidate against *E. tarda* infection.

## 1. Introduction

*Edwardsiella tarda* is a Gram-negative pathogen and the etiological agent of edwardsiellosis, which affects a wide range of marine and freshwater fish [1,2,3], causing enormous economic losses around the world [4,5]. The common method to treat edwardsiellosis is by administration of antibiotics, which would cause environmental pollution and microbial resistance [6]. Vaccines have advantages in safety, environmental friendliness, and long-term efficacy of protection [7], and they have gradually become a mainstream product in disease prevention in aquaculture.

Outer membrane protein C is a porin that resides in the outer membrane of Gram-negative bacteria. Studies in human pathogen *Escherichia coli* and *Salmonella typhi* had showed that OmpC is a major cell surface antigen, and the expression of OmpC during the infection period and its capacity of exhibiting heterologous epitopes on the cell surface make it an attractive candidate antigen in vaccine development [8,9,10,11]. Research has confirmed that OmpC of pathogenic *E. coli*, when in form of subunit vaccine, could induce the production of specific serum antibodies and produce highly protective effects against *E. coli* infection and cross-protection against *Shigella* strains [12]. It was also reported that OmpC of *Salmonella* revealed better protection against virulent *Salmonella* challenge when compared with formalin-killed, whole-cell bacterial vaccine in a bird model [13,14]. Moreover, a *Shigella flexneri* 3a OmpC epitope was recognized by human umbilical cord sera and associated with protective activity, mice immunized with OmpC retained long-lasting protection against a lethal dose of both homologous and heterologous strains of the pathogens [15]. Additionally, OmpC in other Gram-negative bacteria, such as *Aeromonas hydrophila* and *S. enterica* serovar Typhi, has also been reported to have the potential in vaccine development [9,16]. *E. tarda* is a pathogen bacteria that threatens fish, reptiles, amphibians, and humans [17], and great efforts have been directed into vaccine development during the last decade. Though several effective subunit vaccine candidates have been obtained [18], no commercializable subunit vaccine is available. Moreover, frequently outbreaks of edwardsiellosis have highlighted the urgent need to develop a highly protective vaccine. Thus, the values of OmpC in vaccine design against pathogens of human and higher vertebrates may shed light for us to develop a highly protective vaccine against *E. tarda*.

In the present study, OmpC of *E. tarda* was amplified and recombinantly expressed. To test the immunogenicity of OmpC, outer membrane proteins of *E. tarda* were extracted and analyzed by Western blot using flounder (*Paralichthys olivaceus*) anti-rOmpC and anti-*E. tarda* antibodies. Meanwhile, the immune response of flounder after being injected with rOmpC was investigated, including the production of specific serum antibodies, the proliferation of sIg+ lymphocytes and the expression of immune-related genes. Moreover, the immune protective effects were examined by challenging with *E. tarda* in the flounder model.

## 2. Results

### 2.1. Expression and Purification of Recombinant Outer Membrane Protein C

**Figure 1:**
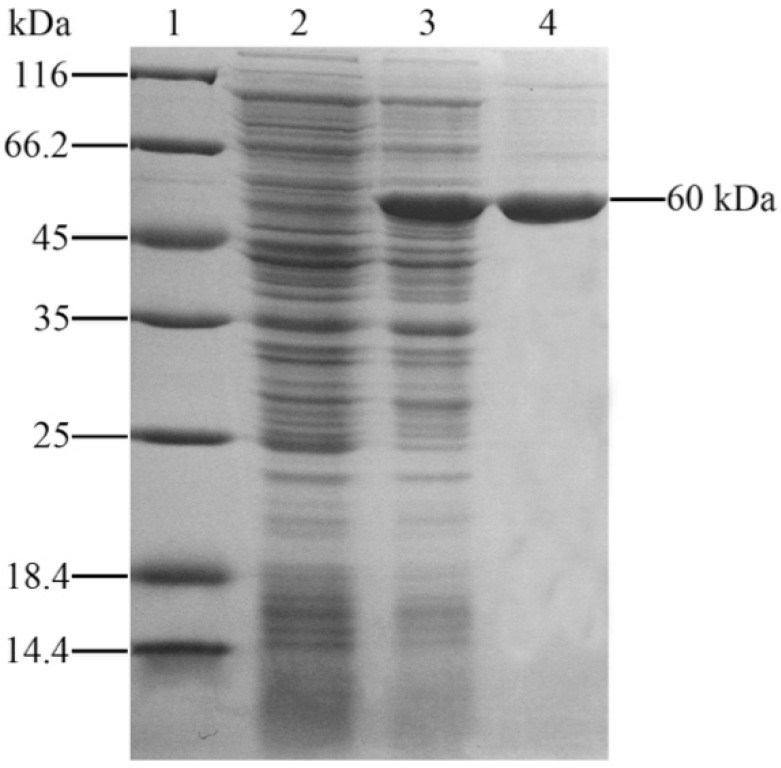
SDS-PAGE analysis of rOmpC. **Lane 1**: molecular mass marker; **Lane 2**: negative control without IPTG induction; **Lane 3**: *E. coli* transfected with pET-32a-OmpC induced with IPTG; **Lane 4**: purified protein of rOmpC.

OmpC with TRX-tag were expressed in *E. coli* BL21 (DE3) with pET-32a system (Merck Millipore, Darmstadt, Germany). Sodium dodecyl sulfate—polyacrylamide gel electrophoresis (SDS-PAGE) revealed that OmpC was successfully expressed after IPTG induction with a distinct band representing about 60 kDa (Figure 1), which was in accordance with the predicted molecular mass of OmpC plus TRX protein mass about 21 kDa [19]. After purification with the Ni^2+^ affinity chromatography, the rOmpC with high purity were obtained.

### 2.2. Analysis of the Immunogenicity of OmpC

**Figure 2:**
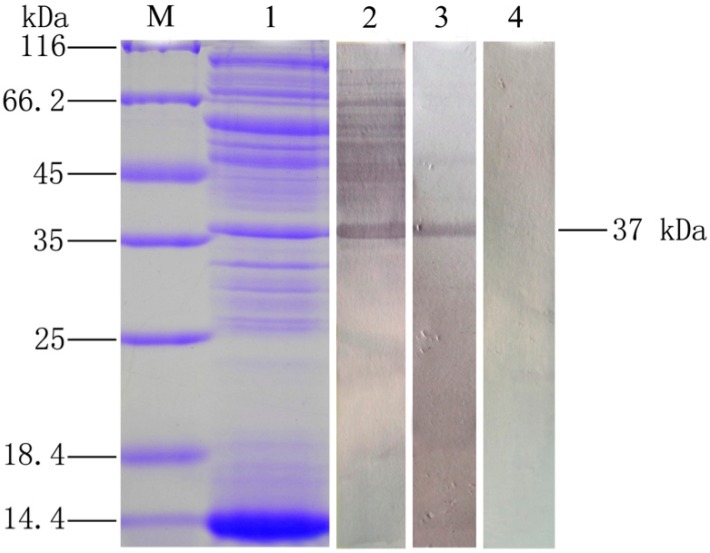
Analysis of the immunogenicity of OmpC by SDS-PAGE and Western blot. **Lane M:** molecular mass marker; **Lane 1**: OMPs extracted from *E. tarda*; **Lane 2**: Western blot analysis using flounder anti-*E. tarda* serum; **Lane 3**: Western blot analysis using flounder anti-rOmpC serum; **Lane 4**: negative control using the serum of healthy founder.

To test the immunogenicity of OmpC, the OMPs of *E. tarda* was extracted and analyzed by Western blot using flounder anti-rOmpC and anti-*E. tarda* antibodies. Interestingly, both of two different antibodies could specifically bind the 37 kDa protein (Figure 2). Mass spectrometric (MS) results showed that the 37 kDa protein matched 4, 2, 3 and 1 peptides with OmpA, OmpC, OmpF, and GAPDH of *E. tarda*, respectively, and all of them obtained high mascot scores. Details of the MS results are given in Table 1.

### 2.3. Flow Cytometric Immunofluorescence Analysis

**Figure 3:**
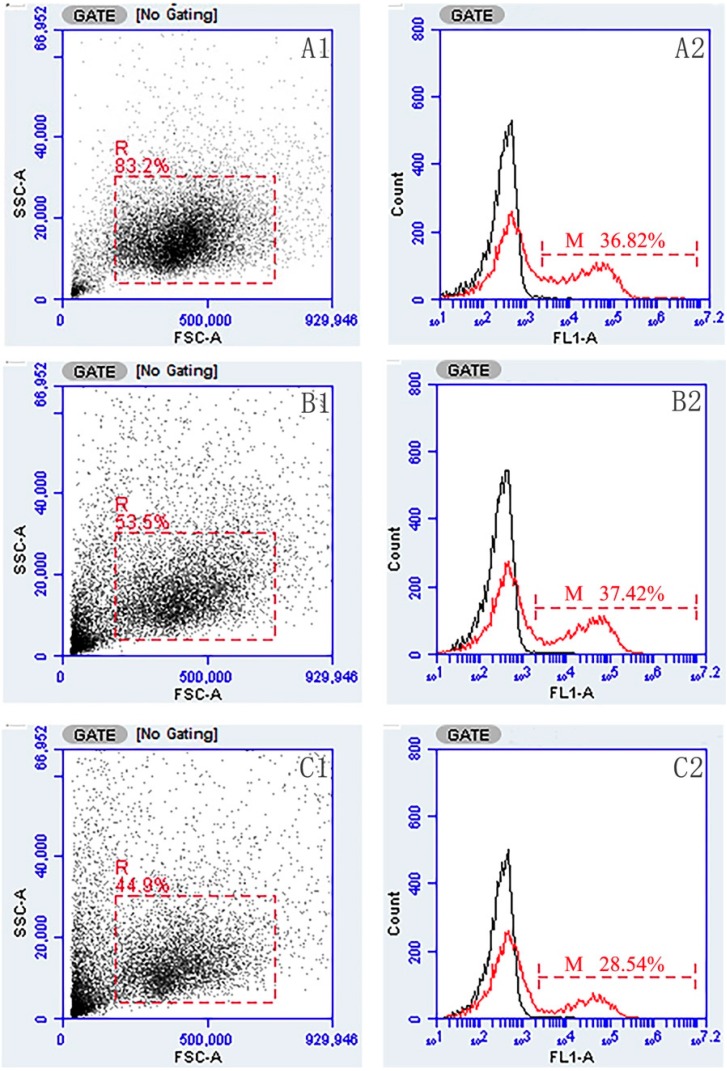
Flow cytometric analysis of lymphocytes reacted with MAb 2D8. **A1**, **B1** and **C1** represented lymphocytes in blood, spleen, and pronephros gated (R) on a forward scatter (FSC) versus side scatter (SSC) dot plot, respectively; **A2**, **B2** and **C2**, rOmpC-injected fish, combined (smoothed) Fluorescein isothiocyanate (FITC) fluorescence histogram of gated lymphocytes (R) showing the percentages of sIg+ lymphocytes (scale of M) in blood, spleen, and pronephros at week 4 after immunization, respectively.

**Figure 4:**
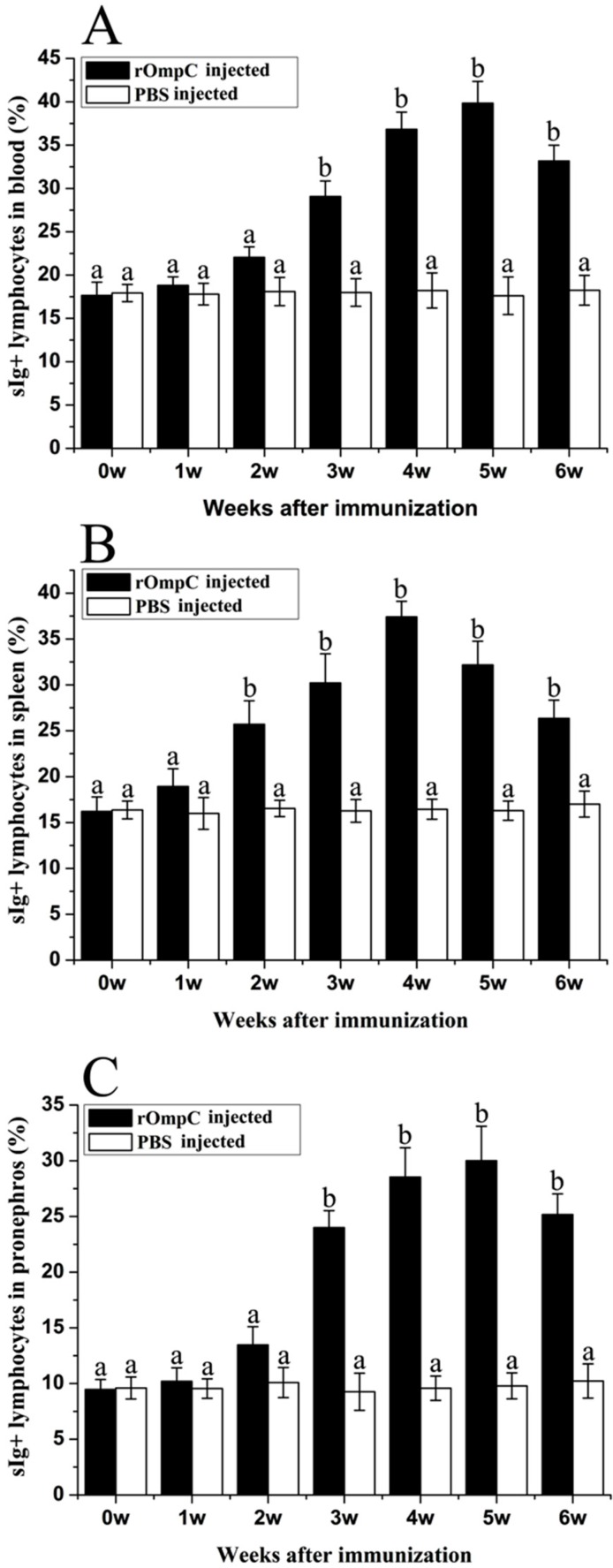
The changes of the levels of sIg+ lymphocytes in blood (**A**), spleen (**B**) and pronephros (**C**) of rOmpC and phosphate-buffered saline (PBS)-injected fish after immunization. Results are expressed as means ± SEM (*n* = 3). Different letters “a” and “b” on the bars represent the statistical significance (*p* < 0.05) compared to each other at same time point.

The gated lymphocytes in Forward scatter/Sideward scatter dot plot and represented fluorescence histograms of rOmpC-injected fish at week 4 after immunization are shown in Figure 3, and the changes of the percentages of sIg+ lymphocytes in different tissues of vaccinated fish were summarized in Figure 4. Three weeks after immunization, the levels of sIg+ lymphocytes in blood and pronephros of rOmpC-injected fish were significantly increased compared with the control group, and the peak levels occurred at week 5 after immunization. However, the level of sIg+ lymphocytes in spleen of rOmpC-injected fish was significantly increased as early as two weeks after immunization compared with the control, and the peak level occurred at week 4 after immunization.

### 2.4. Enzyme Linked Immunosorbent Analysis of Sera Antibodies against E. tarda

**Figure 5:**
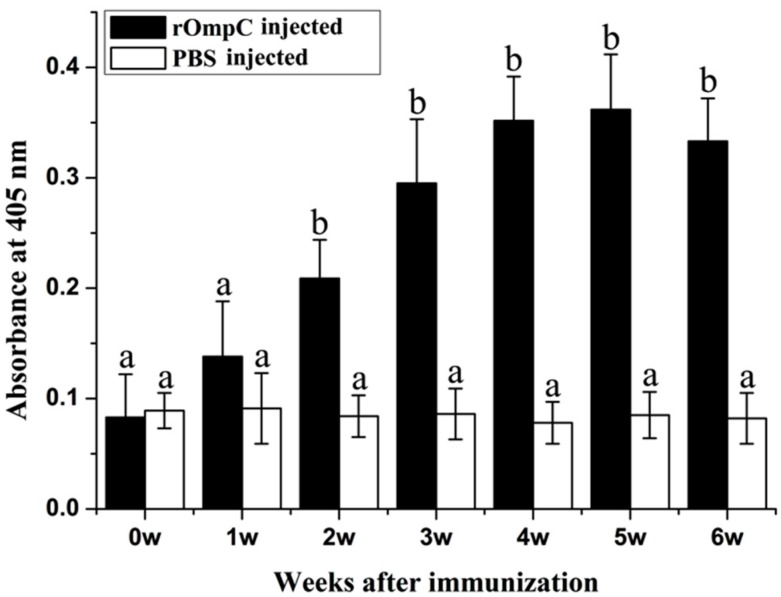
Serum antibodies against *E. tarda* in vaccinated fish. Sera were collected from three fish before immunization and at week 1, 2, 3, 4, 5 and 6 after immunization, serum antibodies against *E. tarda* were determined by ELISA. Results are expressed as means ± SEM (*n* = 3). Different letters “a” and “b” on the bars represent the statistical significance (*p* < 0.05) compared to each other at same time point.

ELISA analysis showed that rOmpC could induce the production of specific serum antibodies against *E. tarda* (Figure 5). The levels of specific serum antibodies induced by rOmpC rapidly increased at week 2, 3, and 4 after immunization, and then reached the peak level at week 5 after immunization. The levels of specific serum antibodies of rOmpC group were significantly higher than the control group except the first week after immunization.

### 2.5. Immune Protective Effects of Recombinant Outer Membrane Protein C

**Figure 6:**
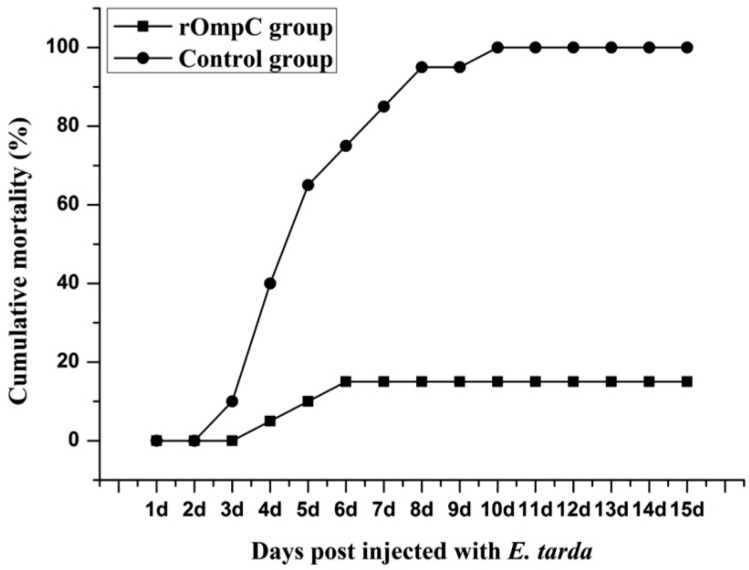
Cumulative mortality of flounder injected with rOmpC or PBS. Six weeks after immunization, all groups were challenged with *E. tarda* of 10^7^ CFU/fish and the mortalities were recorded for 15 d until there were no dead fish found. *E. tarda* was recovered from the liver, spleen and kidney of moribund fish.

Before the challenge experiment, no mortality was found in the rOmpC- or PBS-injected groups and no lesions were found on the skin and internal organs of rOmpC- or PBS-injected flounder when sampled at various time points. While the flounders were challenged with live *E. tarda* at six weeks post vaccination, the fish began to die on day 3 after being challenged, and the cumulative mortality rate of the control group increased rapidly at 1–2 weeks and reached 100% at day 10. However, lower cumulative mortality rates were observed in the fish injected with rOmpC compared with the control group. The cumulative mortality rates of all the experimental groups after being challenged with *E. tarda* are summarized in Figure 6. The results showed that the cumulative mortalities of rOmpC and PBS injected groups were 15% and 100%, respectively, which correspond to an RPS of 85% for rOmpC. The infected flounder showed typical clinical signs of edwardsiellosis, including rectal hernia and abdominal distension, opacity of the eye and exophthalmia, and peripheral hyperemia in mandible lesions. Bacterial tests on the infected flounder also demonstrated that *E. tarda* was the pathogen that caused the death of flounder.

### 2.6. Expression of Immune-Related Genes

**Figure 7:**
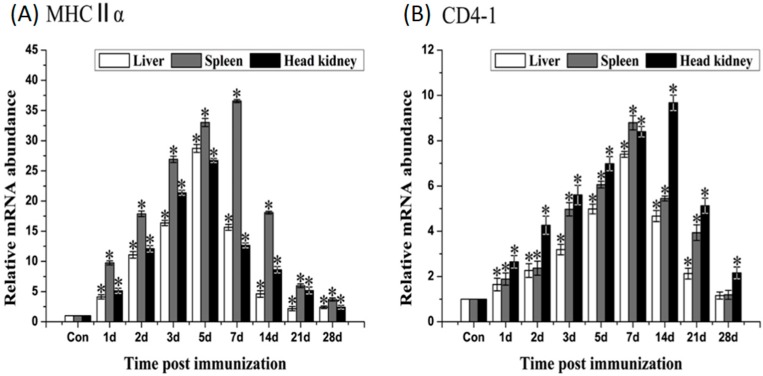
qRT-PCR analysis of the expression of *MHCIIα* (**A**) and *CD4-1* (**B**). The liver, spleen, and head kidney were sampled at 1, 2, 3, 5, 7, 14, 21 and 28 days after immunization. The mRNA level of each immune-related gene was normalized to that of *18S*. For each gene, the mRNA level of the control fish was set as 1. Results are expressed as means ± SEM (*n* = 3). The asterisk indicates the statistical significance compared with the control group (* *p* < 0.05).

**Figure 8:**
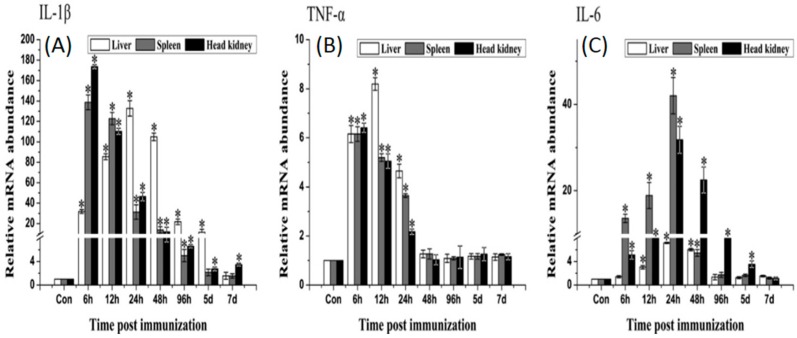
qRT-PCR analysis of the expression of *IL-1β* (**A**), *TNF-α* (**B**) and *IL-6* (**C**). The liver, spleen, and head kidney were sampled at 6 h, 12 h, 24 h, 48 h, 96 h, 5 days and 7 days after immunization. The mRNA levels of each immune-related gene were normalized to those of *18S*. For each gene, the mRNA level of the control fish was set as 1. Results are expressed as means ± SEM (*n* = 3). The asterisk indicates the statistical significance compared with the control group (* *p* < 0.05).

qRT-PCR analysis showed that rOmpC could induce the up-regulation of *MHCIIα*, *CD4-1*, *IL-1β*, *TNF-α* and *IL-6*. *MHCIIα* gene significantly up-regulated and reached the peaks at day 5, day 7 and day 5 after immunization in liver, spleen and head kidney, respectively. *CD4-1* gene was highly induced by rOmpC and the peaks occurred at day 7, day 7 and day 14 after immunization in liver, spleen, and head kidney, respectively (Figure 7). The expression of *IL-1β* and *TNF-α* shared a similar variation, the mRNA levels increased and reached the peaks at 6–24 h after immunization, and then declined. *IL-6* gene significantly up-regulated and reached the peaks at 24–48 h after immunization (Figure 8). However, there was no significant difference in expressions of *TLR2*, *TLR5M*, *IFN-γ*, *MHCIα*, and *CD8α* genes between the rOmpC-injected and the control group.

## 3. Discussion

To test the immunogenicity of OmpC, the OMPs of *E. tarda* were extracted and analyzed by Western blot using flounder anti-rOmpC and anti-*E. tarda* antibodies. Interestingly, both of two specific antibodies could bind the 37 kDa protein. MS results showed that the 37 kDa protein highly matched with OmpC of *E. tarda*. These results may indicate that OmpC was an immunogenic protein, which had high abundance in the surface of *E. tarda*. When flounder was vaccinated with formalin-inactivated *E. tarda*, anti-OmpC antibodies could be effectively induced. Meanwhile, rOmpC could preserve the properties of native OmpC, and antibodies induced by rOmpC could react with native OmpC. Moreover, MS results showed that the 37 kDa protein could also match with OmpA, OmpF, and GAPDH of *E. tarda*, these might be explained that the 37 kDa protein may have more than one homologues. Previous studies showed that a homologue of the 37 kDa protein, glyceraldehyde-3-phosphate dehydrogenase (GAPDH) when in form of recombinant protein could produce immune-protective effects in flounder against edwardsiellosis [20,21]. OmpC and OmpF were homologs and they shared great similarities in the amino sequence with each other [22,23,24]. Additionally, OmpA was highly conserved through evolution and rOmpA could highly protect carp against *E. tarda* infection [25,26]. These may indicate that all of the four proteins may have the potential to be vaccine candidates.

Subunit vaccines are safe and have negligible adverse effects, several researchers have confirmed that subunit vaccines, when in form of recombinant proteins purified from *E. coli* and formulated with certain adjuvants, could induce the production of specific serum antibodies and produce highly protective effects [1,27]. In the present study, flounder was injected with 100 μL of 2 mg/mL rOmpC equally mixed with complete Freund’s adjuvant. During a six-week observation period, no mortality was found in the rOmpC-injected group and no lesions were found on the skin and internal organs of rOmpC-injected flounder when sampled at various time points. Moreover, this dose of rOmpC produced an RPS of 85% against *E. tarda* challenge. All of these results suggested that OmpC was a protective antigen and 2 mg/mL rOmpC was a safe dose. Nevertheless, only one concentration was set in the present study, which might not be the most optimal concentration, so we also believed that a further research was needed to optimize the injection dose by setting a series of concentrations. Meanwhile, the levels of sIg+ lymphocytes and specific antibodies induced by rOmpC were also examined. The results showed that rOmpC could induce the proliferation of sIg+ lymphocytes and the peak levels in blood, spleen, and pronephros occurred at week 5, week 4 and week 5 after immunization. In accordance with the change variation of sIg+ lymphocytes, the specific antibodies increased and reached the peak level at week 5 after immunization. These results may indicate that rOmpC could induce a strong humoral immune response.

It was reported that *MHCI* and *MHCII* genes responsible for encoding cell-surface glycoproteins, which will bind foreign peptides and present self- and non-self peptide to T-cells’ receptors [28,29]. It was also considered that foreign peptides degraded by intracellular pathogens were presented to cytotoxic CD8+ T cells by MHCI molecule [30], and foreign peptides of extra cellular pathogens were presented to CD4+ T cells by MHCII molecule [31]. In our research, *MHCIIα* and *CD4-1* genes significantly up-regulated after immunization, while *MHCIα* and *CD8α* were not induced. Though *E. tarda* was an intracellular pathogen [32], OmpC when in form of recombinant protein may be bound by MHCII and presented to CD4+ T cells, which indicate that the recognition and presentation of rOmpC may involve in MHCII and CD4 pathway. Three important pro-inflammatory cytokines, *IL-1β*, *TNF-α*, and *IL-6*, were also induced by rOmpC. These cytokines play an important role in activating the pro-inflammatory cytokine cascade, in activating the functions of macrophages, and also in activation of the adaptive immune system [33]. TLRs are well known as the key pattern recognition receptors for detecting invading microorganisms [34], however, both *TLR2* and *TLR5M* genes were not induced after vaccination with rOmpC, suggesting that the TLR2 and TLR5 pathways were not activated.

## 4. Materials and Methods

### 4.1. Expression and Purification of Recombinant Outer Membrane Protein C 

Based on the genome sequence of *E. tarda* EIB202 (Genbank No. CP001135.1), the gene sequence encoding OmpC was obtained and the accession number was ETAE3470. Specific primers were designed to amplify the open reading frame (ORF) of OmpC excluding the region coding for its signal peptide. The primers used were all listed in Table 2. The PCR product of OmpC was purified and digested with specific restriction enzymes, and then ligased into pET-32a vector to construct recombinant plasmids. The recombinant plasmid pET-32a-OmpC was transformed into *E. coli* BL21 (DE3). The transformant was cultured in LB medium to a mid-logarithmic phase and induced by adding isopropyl β-d-1-thiogalactopyranoside. His-tagged rOmpC was purified using His Trap™ HP Ni-Agarose (GE healthcare China, Beijing, China) followed by the manufacturer’s instruction. The purified protein was dialyzed for 24 h against phosphate-buffered saline (PBS) and treated with Triton X-114 to remove endotoxin [35]. The protein was analyzed by SDS-PAGE and visualized after staining with Coomassie brilliant blue R-250. The concentrations of proteins were determined using the Bradford method.

### 4.2. Preparation of Flounder Anti-rOmpC and Anti-E. tarda Antibodies

Ten healthy flounder weights of 750 ± 50 g were purchased from local farm and divided randomly into two groups and were immunized via intraperitoneal injection with rOmpC or inactivated *E. tarda* mixed with complete Freund’s adjuvant. The flounder were boosted with the same amount of rOmpC or inactivated *E. tarda* mixed with incomplete Freund’s adjuvant at two weeks after the initial immunization. The fish were sacrificed at two weeks post the last boost, and blood was collected from the caudal veins of the fish. The blood was placed at 4 °C for overnight to allow clotting, and sera were obtained by centrifugation at 3000× *g* for 10 min [36], and then the serum was stored at −20 °C until usage. Before manipulations, the fish were anaesthetized with 100 ng/mL of tricaine methanesulphonate (MS-222, Sigma, St. Louis, MO, USA). For euthanasia, the fish were over-anesthetized with 300 ng/mL of MS-222.

### 4.3. Analysis of the Immunogenicity of OmpC

The OMPs of *E. tarda* were extracted by the method as described [37]. The extracted OMPs were analyzed by SDS-PAGE and transferred onto a PVDF membrane (Merck Millipore, Darmstadt, Germany). The membrane was blocked with PBS containing 4% BSA for 1 h at 37 °C, and incubated with flounder anti-rOmpC serum, anti-*E. tarda* serum or the serum of healthy flounder for 1 h at 37 °C, then washed three times with PBS containing 0.05% Tween-20 (PBST), and then incubated with mAb 2D8 for 1 h at 37 °C, which was a monoclonal antibody against flounder IgM previously produced by our laboratory [38], then washed three times with PBST. Antibody binding was detected with goat-anti-mouse Ig-alkaline phosphatase conjugate (Merck Millipore, Darmstadt, Germany) diluted 1:4000 in PBS for 1 h at 37 °C, and washed three times with PBST. Finally, the bands were stained with freshly prepared substrate solution (100 mM NaCl, 100 mM Tris and 5 mM MgCl_2_, pH 9.5) containing nitroblue tetrazolium (NBT, Sigma, St. Louis, MO, USA) and 5-bromo-4-chloro-3-indolyphosphate (BCIP, Sigma, St. Louis, MO, USA) for 5 min and stopped by washing with distilled water. The immune-reactive proteins were excised from polyacrylamide gels and analyzed by an ABI5800 matrix-assisted laser desorption ionization time-of-flight (MALDI-TOF) assay system (Applied Biosystems, Beverly, MA, USA).

### 4.4. Vaccination and Sampling

Two hundred healthy flounder with body weights of 30 ± 5 g were obtained from a fish farm in Rizhao City of Shandong province, China. The fish were maintained in tanks containing aerated, sand-filtered seawater at 21 ± 0.5 °C for one week prior to processing. Flounder were equally divided into two groups. For rOmpC vaccination, the concentration of rOmpC was adjusted to 2 mg/mL, and one group was intraperitoneally injected with 100 μL rOmpC mixed with complete Freund’s adjuvant at equal volume. The other one was intraperitoneally injected with 100 μL PBS as the control group.

The serum and the lymphocytes in blood, spleen, and pronephros were randomly sampled from three fish in each group before immunization and at week 1, 2, 3, 4, 5 and 6 after immunization. For serum isolation, blood was collected from the caudal veins and allowed to clot overnight at 4 °C. The serum was obtained by centrifugation at 3000× *g* for 10 min and stored at −20 °C until usage. For qRT-PCR, the liver, spleen, and head kidney were randomly collected from three fish in each group at 6 h, 12 h, 24 h (1 day), 48 h (2 days), 72 h (3 days), 96 h (4 days), 5 days, 7 days, 14 days, 21 days and 28 days after immunization. Tissues were placed in a sample protector (Baosheng, Dalian, China) and stored at −80 °C until usage. Before manipulations, the fish were anaesthetized or over-anaesthetized with MS-222 as before.

### 4.5. Flow Cytometric Immunofluorescence Analysis

The lymphocytes in blood, spleen, and pronephros of vaccinated flounder were isolated according to the technique as described in our previous study [38]. The lymphocytes in PBS were counted and diluted to 1 × 10^6^ cells/mL, then incubated with mAb 2D8 (1:3000 diluted in PBS) for 1 h at 37 °C. Subsequently, cells were washed three times with PBS containing 5% (*v*/*v*) Newborn Calf Serum, then incubated with goat-anti-mouse Ig-FITC (1:256 diluted in PBS, Sigma) for 1 h at 37 °C, and washed again. After that, the cell suspensions were analyzed with an Accuri C6 cytometer (BD Accuri™, Piscataway, NJ, USA).

### 4.6. Detection of the Serum Antibodies against E. tarda by ELISA

Wells of flat-bottom microplates (96-wells, Costar) were coated overnight with 100 μL/well of *E. tarda* (10^8^ CFU/mL) at 4 °C. The wells were washed three times with PBST and then blocked with 3% BSA in PBS for 1 h at 37 °C. After washing, the serum (1:100 diluted in PBS) sampled from vaccinated fish at different times were added 100 μL per well and incubated for 2 h at 37 °C. Following washing, 100 μL mAb 2D8 (1:3000 diluted in PBS) was added for serum antibody detection. After incubation at 37 °C for 1 h and washing, 100 μL goat-anti-mouse Ig-alkaline phosphatase conjugate (Sigma) diluted 1:5000 in PBS was added and incubated for 1 h at 37 °C. After the last washing, 100 μL 0.1% (*w*/*v*) p-nitrophenyl phosphate (pNPP, Sigma, USA) in 50 mM carbonate-bicarbonate buffer (pH 9.8) containing 0.5 mM MgCl_2_ was added to each well and incubated at room temperature for 30 min in the dark. The reaction was stopped by adding 50 μL per well of 2 M NaOH and absorbance was measured with an automatic ELISA reader (TECAN, Männedorf, Switzerland) at 405 nm.

### 4.7. Analysis of the Expression of Immune-Related Genes by qRT-PCR

Total RNA was extracted from the liver, spleen, and head kidney of vaccinated fish using trizol reagent according to the manufactures’ instruction and measured by a Nanodrop 8000 Spectrophotometer (Thermo Scientific, Waltham, MA, USA). Single-strand cDNA was synthesized from 2 μg total RNA using PrimeScript™ RT-PCR Kit (Baosheng, Dalian, China) according to the manufactures’ instruction. qRT-PCR was carried out using SYBR GreenI Master (Roche, Basel, Switzerland) in a LightCycler^®^ 480 II Real-Time System (Roche, Basel, Switzerland). Each assay was performed in triplicate with *18S* gene as the internal control. All dates were analyzed relative to the *18S* gene by the 2^−∆∆*C*t^ method, then the difference in the vaccinated and control groups were employed to assess changes in the expression of genes. The primers for *TLR2*, *TLR5M*, *MHCIα*, *MHCIIα*, *CD4-1*, *CD8α*, *IL-1β*, *IL-6*, *IFN-γ*, *TNF-α* and *18S* were listed in Table 2.

### 4.8. Challenge

Forty vaccinated fish were randomly selected from each vaccinated group for challenge test at week 6 after immunization. The *E. tarda* used for challenge was cultured in LB media at 30 °C for 24 h. The fish was intraperitoneally injected with a dose of 100 μL per fish containing 1 × 10^7^ CFU live *E. tarda*. Mortalities were monitored over a period of 15 days after the challenge, and relative percentage of survival rate (RPS) was calculated as previously described [39].

### 4.9. Statistical Analysis

The statistical analysis was performed using Statistical Product and Service Solution (SPSS) software (Version 20.0; SPSS, IBM, Armonk, NY, USA), differences were analyzed with one-way analysis of variance (ANOVA) and the results were expressed as mean ± SEM. In all cases, the significance level was defined as *p* < 0.05.

## 5. Conclusions

In conclusion, OmpC of *E. tarda* is an immunogenic surface protein, which could induce strong innate immune response and humoral immune response in flounder and finally evoke highly protective effects against *E. tarda* challenge when in the form of a recombinant protein. This indicates that OmpC is a promising vaccine candidate against *E. tarda* infection.

## Figures and Tables

**Table 1:** Mass spectrometric results of the 37 kDa protein.

Number	Protein Name	Accession No.	Theoretical pI	Theoretical *M*_W_ (Da)	Mascot Score /No. of Match Peptides	Protein Coverage (%)
1	Outer membrane protein A	gi|269138621	8.28	38057	350/4	19
2	Outer membrane protein C	gi|387867288	5.26	40872	265/2	11
3	Outer membrane porin F protein	gi|269138593	5.03	40058	429/3	19
4	Glyceraldehyde-3-phosphate dehydrogenase	gi|550649563	6.60	35567	196/1	9

**Table 2:** Primers used in this study.

Primer No.	Primer Name	Primer Sequence	Source
1	OmpC-F	5′-CGAGCTC ATGATGAATAAAATCCGCTC-3’	ETAE3470
2	OmpC-R	5’-CCGCTCGAGTTAGAACTTATAGTTCAGCATGG-3’	
3	18sRNA-F	5’-GGTCTGTGATGCCCTTAGATGTC-3’	EF126037
4	18sRNA-R	5’-AGTGGGGTTCAGCGGGTTAC-3’	
5	TLR2-F	5’-CTGCGGTGTAGCGTTAGTGG-3’	AB109393
6	TLR2-R	5’-CGAAGGCATCATAGGAAAGC-3’	
7	TLR5M-F	5’-TCCAGCATCATTACCAA-3’	AB562152
8	TLR5M-R	5’-TCATACCCAAGTTAGCG-3’	
9	IL-1β-F	5’-CTGTCGTTCTGGGCATCAAA-3’	AB720983
10	IL-1β-R	5’-AACAGAAATCGCACCATCTCACT-3’	
11	TNFα-F	5’-GTCCTGGCGTTTTCTTGGTA-3’	AB040448
12	TNFα-R	5’-CTTGGCTCTGCTGCTGATTT-3’	
13	IFN-γ-F	5’-TGTCAGGTCAGAGGATCACACAT-3’	AB435093
14	IFN-γ-R	5’-GCAGGAGGTTCTGGATGGTTT-3’	
15	IL-6-F	5’-CTCCGCAATGGGAAGGTG-3’	DQ267937
16	IL-6-R	5’-GATGGATGGGTGGAATAA-3’	
17	MHCΙα-F	5’-AGACCACAGGCTGTTATCACCA-3’	AB126921
18	MHCΙα-R	5’-TCTTCCCATGCTCCACGAA-3’	
19	MHCΙΙα-F	5’-ACAGGGACGGAACTTATCAACG-3’	AY997530
20	MHCΙΙα-R	5’-TCATCGGACTGGAGGGAGG-3’	
21	CD4-1-F	5’-CCAGTGGTCCCCACCTAAAA-3’	AB643634
22	CD4-1-R	5’-CACTTCTGGGACGGTGAGATG-3’	
23	CD8α-F	5’-CCTCTCCCCATACATTGATTCC-3’	AB082957
24	CD8α-R	5’-CCGAGCTTTGCTGAAGGACTT-3’	

The underlined letters represent the restriction enzyme sites.
